# Identifying Differentially Expressed MicroRNAs, Target Genes, and Key Pathways Deregulated in Patients with Liver Diseases

**DOI:** 10.3390/ijms21197368

**Published:** 2020-10-06

**Authors:** Maryam Gholizadeh, Sylwia Szelag-Pieniek, Mariola Post, Mateusz Kurzawski, Jesus Prieto, Josepmaria Argemi, Marek Drozdzik, Lars Kaderali

**Affiliations:** 1 Institute of Bioinformatics, University Medicine Greifswald, Felix-Hausdorff-Str. 8, 17475 Greifswald, Germany; ma.gholizade@gmail.com; 2 Department of Experimental and Clinical Pharmacology, Pomeranian Medical University, 70-111 Szczecin, Poland; sylwia.szelag@pum.edu.pl (S.S.-P.); mkurz@pum.edu.pl (M.K.); 3 Department of General and Transplantation Surgery, County Hospital, 70-111 Szczecin, Poland; mariolapost@wp.pl; 4 Center for Applied Medical Research, University of Navarra, 31008 Pamplona, Spain; jprieto@unav.es; 5 Liver Unit. Clinical Universidad de Navarra, 31008 Pamplona, Spain; jargemi@unav.es

**Keywords:** liver disease, microRNA, regulatory networks

## Abstract

Liver diseases are important causes of morbidity and mortality worldwide. The aim of this study was to identify differentially expressed microRNAs (miRNAs), target genes, and key pathways as innovative diagnostic biomarkers in liver patients with different pathology and functional state. We determined, using RT-qPCR, the expression of 472 miRNAs in 125 explanted livers from subjects with six different liver pathologies and from control livers. ANOVA was employed to obtain differentially expressed miRNAs (DEMs), and miRDB (MicroRNA target prediction database) was used to predict target genes. A miRNA–gene differential regulatory (MGDR) network was constructed for each condition. Key miRNAs were detected using topological analysis. Enrichment analysis for DEMs was performed using the Database for Annotation, Visualization, and Integrated Discovery (DAVID). We identified important DEMs common and specific to the different patient groups and disease progression stages. hsa-miR-1275 was universally downregulated regardless the disease etiology and stage, while hsa-let-7a*, hsa-miR-195, hsa-miR-374, and hsa-miR-378 were deregulated. The most significantly enriched pathways of target genes controlled by these miRNAs comprise p53 tumor suppressor protein (TP53)-regulated metabolic genes, and those involved in regulation of methyl-CpG-binding protein 2 (MECP2) expression, phosphatase and tensin homolog (PTEN) messenger RNA (mRNA) translation and copper homeostasis. Our findings show a novel panel of deregulated miRNAs in the liver tissue from patients with different liver pathologies. These miRNAs hold potential as biomarkers for diagnosis and staging of liver diseases.

## 1. Introduction

The liver is a major solid organ and is involved in a number of key metabolic processes. Damaged or reduced functional capacity of this organ can result in jaundice, hypoglycemia, deficient blood clotting, protein malnutrition, increased risk of infection, impaired lung and kidney function, fluid retention, and fatigue [1]. Liver disease here refers to a wide spectrum of both acute conditions caused by various injurious agents, such as viruses, toxins, alcohol, and pharmacological agents, which can lead to chronic inflammation and liver cirrhosis. The major causes of liver cirrhosis are hepatitis C and B infections, alcoholic liver disease, nonalcoholic steatohepatitis and hemochromatosis [2]. Primary liver cancer, especially hepatocellular carcinoma, is part of the natural history of cirrhosis, regardless of the etiology. Although severe liver disease is often asymptomatic or linked to vague symptoms, such as malaise and fatigue, it results in significant short-term morbidity and progressively impairs an individual’s quality of life. Liver failure of any degree, once present, is associated with a significantly increased risk of premature death [3]. Current management of liver cirrhosis has improved remarkably in recent years; however, most patients, for which the etiology is not eliminated, still progress to advance liver fibrosis and ultimately to decompensated cirrhosis, with a fatal outcome without liver transplant, associated with a high healthcare economic burden. Thus, further investigation to identify the most important biomarkers for early diagnosis, staging, and prevention, as well as for novel target discovery, is still needed.

MicroRNAs (miRNAs) represent a new class of highly conserved, endogenous, single-stranded, and small noncoding RNAs (~22 nucleotides) that are involved in the regulation of gene expression by directly degrading the messenger RNA (mRNA) or blocking protein translation by binding to the 3′ untranslated region of the target mRNAs. After the discovery of the first miRNA, considerable efforts have been made to understand miRNA biology [4]. On the basis of findings related to the role of miRNAs in diseases progression, miRNAs are regarded as attractive tools and biomarkers for new therapeutic approaches. Since miRNAs are highly stable in body fluids and also play a crucial role in the pathophysiology of liver injury, they represent promising targets for the development of strategies to identify, prevent, or treat liver diseases. We know that miRNA expression signatures can be highly tissue- and disease-specific [5]. Although passenger miRNA (miRNA*) was originally described as a nonfunctional strand that is cleaved and degraded by Ago2, there is growing evidence of its possible regulatory activity. It was reported that the miRNA/miRNA* ratio may vary between multiple tissue samples, probably due to changes in miRNA* degradation [6], and, in some cases, even strand dominance may change [7]. For example, hsa-let-7a* was downregulated in fibroblasts infected with varicella zoster virus [8], and mir-374b* was upregulated during antibody-mediated renal graft rejection [9]. Approximately 70% of all miRNAs are expressed in the liver. Evidence is emerging that miRNA expression profiles are distinct between liver diseases and normal tissue. The most abundant miRNA in the liver is miR-122, which is essential for proper hepatocyte proliferation and differentiation. Its dysregulation has been shown in drug-induced liver injury [10] and hepatitis C virus (HCV)-induced necroinflammation and fibrosis [11]. miR-29 and miR-21 have a role in liver fibrogenesis [12], and miR-221 plays a role in liver regeneration and protection in a model of cluster of differentiation 95 (CD95) ligand (CD95L) induced fulminant liver failure [13,14]. Deregulation of miR-223 expression has been observed in various types of liver diseases [15]. Finally, the downregulation of miR-122a and miR-16a and the upregulation of miR-328 and miR-299-5p have been described in primary biliary cholangitis (PBC) [16]. Despite this evidence, a comprehensive comparative study that analyzes the relative abundance of relevant miRNAs in liver tissue with regard to both etiology and cirrhosis progression is still lacking. The present study aimed to investigate the key miRNAs in a selection of liver diseases compared to normal liver tissue, as well as their association stage of cirrhosis progression according to the Child–Pugh score (CPS).

Currently, there are a number of methods for miRNA profiling: hybridization (microarray), small RNA sequencing (miRNAseq), and reverse-transcription quantitative PCR (RT-qPCR) [17]. In order to identify possible roles of miRNAs and to better understand the molecular mechanisms of liver diseases, we integrated miRNA and mRNA analysis. Therefore, we used a computational method to derive a miRNA–gene differential regulatory networks (MGDRN) for data analysis. First, miRNA expression profiles in livers from patients with different pathology and functional state were obtained using real-time PCR. Next, we identified differentially expressed miRNAs (DEMs); subsequently, their target genes were derived using a comprehensive database search. To evaluate the interactive relationships between differentially expressed miRNA target genes, protein–protein interaction (PPI) networks were constructed and modules were screened. The MGDRN was constructed by considering predicted miRNA–target gene interactions and their regulatory features. Then, we assayed the topological parameters of the network and found key etiology-specific miRNAs. Finally, we performed functional enrichment analysis of DEM target genes, using the Database for Annotation, Visualization, and Integrated Discovery (DAVID) and Gene Ontology (GO). We successfully identified important DEMs common and specific to the different patient groups and disease progression stages. The results of this work could provide clues for the development of new strategies of early detection and in-depth mechanistic studies of specific miRNA targets in liver diseases.

## 2. Results

### 2.1. Identification of DEMs and Target Gene Prediction along Liver Cirrhosis Staging

**Figure 1:**
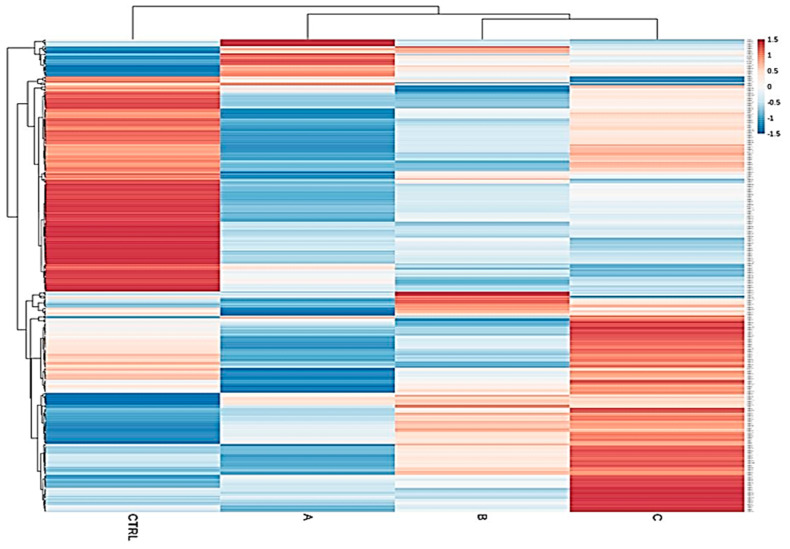
Heatmap of total samples based on the Child–Pugh score (A, B and C). A clear separation of upregulated and downregulated microRNA (miRNA) profiles of the control group from the diseased cohorts emerged, confirming that miRNA expression patterns between these classes and the normal group are strongly different.

We determined, using RT-qPCR, the expression of 472 miRNAs in 125 explanted livers from subjects with six different liver pathologies, and from normal, functional control livers (Table S1). Liver diseases considered were hepatitis C (HCV, *n* = 23), primary biliary cholangitis (PBC, *n* = 14), primary sclerosing cholangitis (PSC, *n* = 8), alcoholic liver disease (ALD, *n* = 22), Wilson’s disease (WD, *n* = 9) and autoimmune hepatitis (AIH, *n* = 16). As shown in Table 1, 26.66% of patients were CPS class A, 37.77% were class B, and 35.55% were class C. A clear separation of upregulated and downregulated miRNA profiles of the control group from the diseased cohorts emerged, confirming that miRNA expression patterns between these classes and the normal group are strongly different (Figure 1). The result of ANOVA indicated that the expression of 123 miRNAs was significantly different in the CPS class A group compared to normal, of which only nine were upregulated and 114 were downregulated in CPS A patients. When comparing normal livers to the CPS class B group, we identified 136 DEMs, including 38 upregulated and 98 downregulated miRNAs. Within the CPS class C group, we found 134 DEMs, of which 62 were upregulated and 72 were downregulated (Table S2). As shown in Figure 1, this specific progression-related miRNA pattern suggested that the magnitude of miRNA downregulation is higher in early stages of liver damage (CPS A), while the set of upregulated miRNAs increases with disease severity (CPS C). Our results of the statistical analysis further show that the expression of 106 and 73 miRNAs was significantly different in females and males, respectively, between the liver pathologies and the controls (Table S3).

### 2.2. Identification of Modules from the PPI Networks

**Figure 2:**
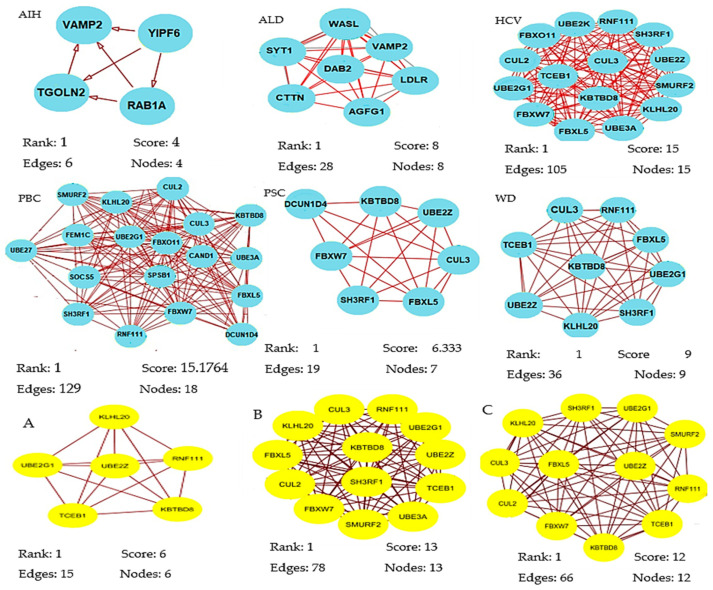
Significant modules of target genes of the differentially expressed miRNAs (DEMs) for each group were obtained from the PPI network using the MCODE plug-in. Rank is based on the cluster’s computed score and is used to identify the clusters within each result. Cluster 1 is the highest ranked cluster in a given result and, thus, at the top of the list. Nodes and edges is are simple enumeration of the cluster’s members and their interconnections. Shown is the top cluster from each condition tested.

A total of 320 miRNAs (182 downregulated; 138 upregulated) were significantly differentially expressed in the liver pathologies compared to the control group (significance level α = 0.05). There were 123 DEMs in HCV patients (down: 63, up: 60), 87 in ALD (down: 49, up: 38), 79 in PBC (down: 39, up: 40), 71 in WD (down: 34, up: 37), 52 in PSC (down: 29, up: 23) in PSC, and 29 DEMs in AIH (down: 18, up: 11). The results of the statistical analysis also showed several DEMs (ALD, 41; AIH, 22; HCV, 46; PBC, 24) in patients who scored 9 and 10 points in the Child–Pugh classification (selected in order to compare livers of different pathology at the same classification stage) as compared to the controls (Table S4). We next obtained gene targets of differentially expressed miRNAs from the miRDB database (Table S5). According to the target score, 845 non-redundant target genes of the 320 miRNAs were selected. On the basis of the information from the Search Tool for the Retrieval of Interacting Genes (STRING) database, a PPI network was constructed of the target genes of the DEMs for each group (Supplementary File S6). Summary information on each network is presented in Table 2. From each network, we then inferred the significant modules for each group using the Molecular Complex Detection (MCODE) plug-in (Table S7, Figure 2). The significant module of AIH included four nodes (RAB1A, YIPF6, VAMP2, TGOLN2) and six edges. 

Biological process enrichment analysis showed that these hub genes were mainly associated with vesicle-mediated transport (Molecular Signatures Database; MSigDB). The module of the ALD network included eight nodes (AGFG1, SYT1, VAMP2, WASL, DAB2, LDLR, SYNJ1, CTTN) engaged in clathrin-mediated endocytosis (MSigDB) and 28 edges involved in biological regulation, developmental process, metabolic process, and immune system process. The module of the PBC network consisted of 18 nodes (SMURF2, KLHL20, CUL2, CUL3, KBTB8, FBXO11, UBE2G1, FEM1C, UBE27, CAND1, UBE3A, SOCS5, SPSB1, FBXL5, SH3RF1, FBXW7, RNF111, FBXW7, DCUN1D4) involved in protein modification by small protection conjugation or removal (16/18 genes, MSigDB) and 129 edges that were enriched in response to stimulus and metabolic process. The module of the PSC network included seven nodes (SH3RF1, FBXL5, UBE2Z, KBTBD8, DCUN1D4, CUL3, FBXW7) participating in ubiquitin-proteasome degradation (6/7 genes, MSigDB) and 19 edges that were enriched in biological regulation, metabolic process, and cellular process. The module of the WD network consisted of nine nodes (CUL3, RNF111, FBXL5, TCEB1, KBTBD8, UBE2G1, UBE2Z, KLHL20, SH3RF1) involved in ubiquitin-proteasome degradation and class I MHC-mediated antigen processing presentation (MSigDB) and 36 edges that were enriched in metabolic process.

The most significant module from the PPI network of HCV consisted of 15 nodes (RNF111, UBE2K, FBXO11, SH3RF1, CUL3, CUL2, UBE2Z, TCEB1, SMURF2, UBE2G1, KBTBO8, FBXW7, KLHL20, FBXL5, UBE3A) engaged in ubiquitin-proteasome degradation and class I MHC-mediated antigen processing presentation (MSigDB) and 105 edges that participated in response to stimulus and cellular process. The most significant module of PPI network of A, B, and C Child–Pugh score group consisted of six, 13 and 12 nodes, respectively (Figure 2). Functional annotation showed that these nodes were mainly involved in biological processes associated with metabolic processes, biological regulation, cellular processes, and response to stimuli.

### 2.3. Construction of MGDRN for Different Liver Diseases

**Figure 3:**
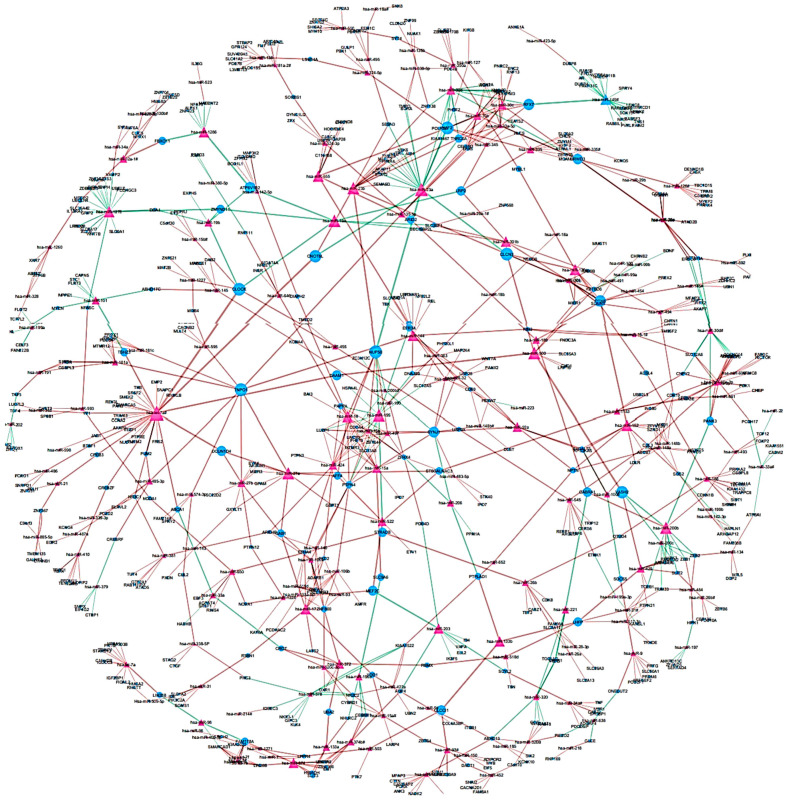
An Overview of the miRNA–target gene differential regulatory network (MGDRN); the purple triangular and blue circular nodes represent miRNAs and target genes, respectively. The size of nodes represents the degrees of nodes in the network. The brown and green edges in the MGDRN represent downregulation and upregulation involving liver disease versus normal conditions, respectively.

**Figure 4:**
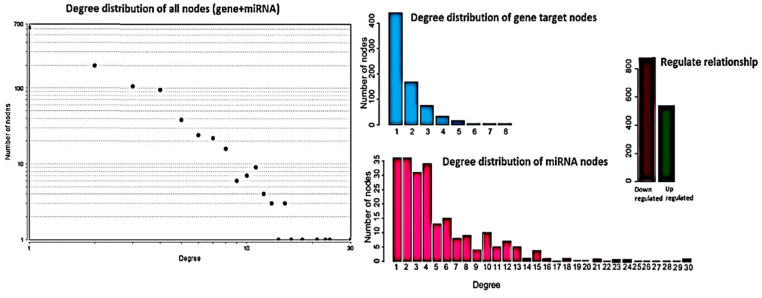
Topological features of the miRNA–target gene differential regulatory network (MGDRN). The degrees of all nodes and miRNAs ranged from 1–30, and the degrees of messenger RNA (mRNA) ranged from 1–8. The total numbers of down- and upregulated relationships in the MGDRN were 879 and 541, respectively.

To identify differentially regulated miRNA–target gene relationships, we constructed the MGDRN using interactions included in the miRDB database. We found 1420 interactions between 320 miRNAs and 845 target genes (Figure 3). The nodes in the network represent DEMs or related target genes, and the edges represent the relationship between molecules. In total, there were 1165 nodes with 879 downregulated relationships (brown edges) and 541 upregulated relationships (green edges) (Figure 3, Table S8). We then analyzed the topological features of the MGDRN. For each node, the degree, betweenness centrality, and closeness centrality were calculated. The degrees of all miRNAs ranged from 1–30, whereas the degrees of mRNAs ranged from 1–8 (Figure 4). Because of a close relationship between miRNAs and the liver, it is not surprising that many miRNAs are involved in different liver diseases. To more deeply investigate the most important miRNAs involved in each liver pathology, we constructed an MGDRN for each specific condition, with topological features shown in Table 3. Hence, hub miRNAs for every condition, according to the highest score in all topological features (degree, betweenness centrality, and closeness centrality), were identified. Table 4 shows key miRNAs involved in different liver pathologies.

In all of the networks except for the ALD network, hsa-miR-1275 was one of the key miRNAs. Furthermore, the key miRNAs included hsa-miR-222, hsa-let-7a*, hsa-miR-1260, hsa-miR-21, and hsa-miR-1275, which are jointly involved in AIH. Results of our topological analysis also show that hsa-miR-195, hsa-miR-182, and hsa-miR-23a are highly expressed in ALD, indicating that expression of these miRNAs may be associated with its progression. hsa-miR-1227, hsa-miR-1275, and hsa-miR-148a were significantly downregulated and hsa-miR-374 was upregulated in livers of patients with hepatitis C. In liver tissues of patients with PBC, we identified hsa-miR-320, hsa-miR-1227, hsa-miR-1275, and hsa-miR-148a as expressed at lower levels than in normal tissue, whereas hsa-miR-374b* was upregulated.

Overall, four hub miRNAs (hsa-miR-23b, hsa-miR-378, hsa-miR-1275, hsa-miR-148a) of PSC patients were downregulated compared to healthy tissue. To detect the key miRNAs, topological features of all nodes of the MGDRN were ranked according to maximum degree, betweenness centrality, and closeness centrality, and we finally selected five miRNAs (hsa-let-7a*, hsa-miR-23a, hsa-miR-195, hsa-miR-23b, hsa-miR-16) that were among the top miRNAs in all topological features. As shown in Table 4, hsa-let-7a* and hsa-miR-23b were the most significantly upregulated, and hsa-miR-23a, hsa-miR-195, and hsa-miR-1275 were the most significantly downregulated microRNAs. Network analyses of Wilson’s disease patients showed that two miRNAs, hsa-miR-1275 and hsa-miR-21, appear to have a specific role in the regulation of gene targets involved in WD.

On the other hand, analysis of the miRNA–gene differential regulatory network of all patients according to the Child–Pugh score classification identified seven hub miRNAs (hsa-miR-1275, hsa-miR-29a, hsa-miR-320, hsa-miR-1227, hsa-miR-378, hsa-miR-21, and hsa-miR-182), which could serve as biomarkers for prognosis, diagnosis, and treatment of different stages of liver diseases. All patients with Child–Pugh score A and B had a significant decrease in expression of hsa-miR-1275 and hsa-miR-320. Patients with the advanced stage of liver disease (Child–Pugh score C) showed a higher expression of hsa-miR-21 and hsa-miR-182 compared to the lower Child–Pugh score groups.

### 2.4. Functional and Pathway Enrichment Analysis of Hub miRNA Target Genes

**Figure 5:**
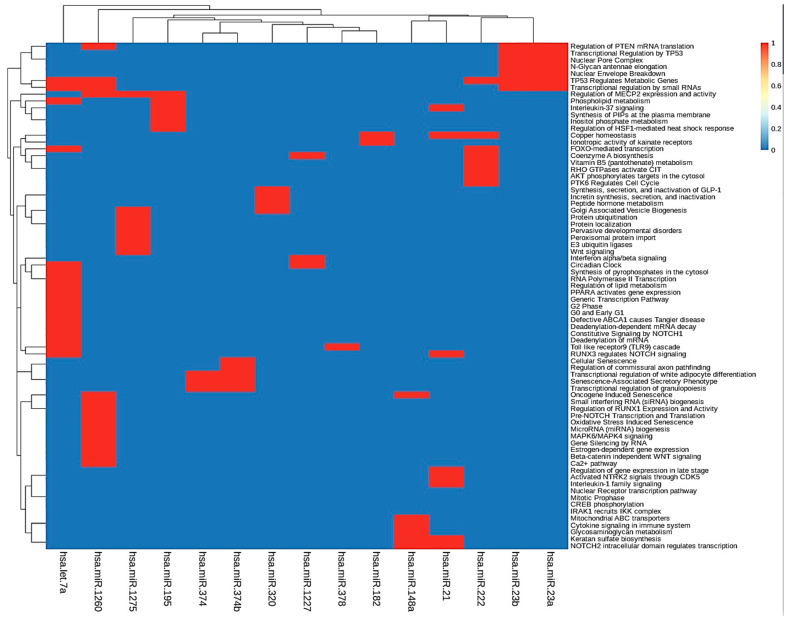
KEGG pathway enrichment analyses were performed to identify miRNA dysregulated pathways using their target dysregulated genes in the MGDRNs. The heatmap of the pathways as a function of miRNAs is shown; bidirectional hierarchical clustering was performed using the R package.

To further investigate the function of the identified target genes of differentially expressed miRNAs, DAVID analysis was performed to analyze functional and pathway enrichment. First, we determined pathways controlled by hub miRNAs in the MGDRNs using their target genes. The topological analysis of the MGDRNs of all studied liver pathologies led to the identification of 15 key miRNAs (eight downregulated; seven upregulated). Pathway analysis was conducted on the 195 mRNAs predicted as targets of these 15 miRNAs, and 74 significantly enriched Kyoto Encyclopedia of Gene and Genomes (KEGG) pathways (*p* ≤ 0.01) were identified (Table S6). Notably, as our results show, a single miRNA can regulate multiple pathways and a single pathway can be regulated by multiple miRNAs. Figure 5 illustrates obvious modules in the heatmap plot. The results show that four miRNAs (hsa-let-7a*, hsa-mir-1260, hsa-mir-23b, and hsa-mir-23a) are involved in the regulation of the function of 35 pathways. Most of these pathways are related to transcriptional regulation by small RNAs, regulation of mRNA translation, regulation of metabolic genes, *N*-glycan antennae elongation, nuclear envelope breakdown, miRNA biogenesis, regulation of lipid metabolism, and MAPK6/MAPK4 signaling. These four miRNAs may play crucial roles by controlling related pathways involved in liver diseases.

Impaired clathrin-mediated internalization was documented in ALD. Dysfunctional clathrin-mediated endocytosis from the sinusoidal surface, produced by ethanol exposure, alters numerous hepatic processes, such as trafficking of asialoglycoproteins and their receptor, and transferrin via the transferrin receptor or internalization of numerous cytokines and growth factors (TGF-a, TNF-a, IL-6, epidermal growth factor, insulin, growth hormone) [18]. The Wilson protein, a copper-transporting P-type ATPase, when mutated, becomes trapped in the endoplasmic reticulum and is presumed to be the molecular basis of disease [19].

The ubiquitin-proteasome degradation of bile acid transporter, i.e., apical sodium-dependent bile acid transporter (ASBT) expressed in the cholangiocyte apical membrane, may be implicated in the pathogenesis of PBC and PSC [20]. Likewise, ubiquitination, which facilitates degradation of bile salt export pump (BSEP) and multidrug resistance-associated protein 2 (MRP2), expressed at the canalicular membrane of hepatocytes, can play a role in PBS and PSC [21]. In hepatitis C, ubiquitylation and proteasomal degradation of retinoblastoma protein (pRb) with concomitant E2F transcription factor release and cellular proliferation can contribute to the disease progression and development of hepatocellular carcinoma [22]. Our results are also in line with a study, which, after proteomic analysis, revealed a possible implication of dysfunctional vesicle-mediated transport in the pathogenesis of AIH [23].

When considering the Child–Pugh classification, the results of KEGG pathway analysis showed 38 significant pathways (*p* ≤ 0.01) for 79 mRNAs predicted as targets of the seven hub miRNAs. Performing pathway analysis showed that the downregulated hub miRNAs (hsa-miR-1275, hsa-miR-1227, hsa-miR-378, hsa-miR-320, hsa-miR-29a) were mainly enriched in regulation of MECP2 expression and activity, epigenetic regulation of gene expression, degradation of the extracellular matrix, and peroxisomal protein import.

## 3. Discussion

Some DEMs emerged as commonly altered in patients with liver disease and associated with disease progression: hsa-miR-122-5p, hsa-miR-122-3p, hsa-miR-483-5p, hsa-miR-1274B, hsa-miR-345, hsa-miR-193a-5p, hsa-miR-193B, hsa-miR-1275, and hsa-miR-139-5p. It is well documented that hsa-miR-122 is one of the most well-known liver-specific miRNAs that plays a central role in progression of liver disease and is involved in the regulation of gene networks and pathways in HCV infection [24].

The result of exploring significant modules showed four nodes in AIH, eight nodes in ALD, 15 nodes in HCV, 18 nodes in PBC, seven nodes in PSC, and nine nodes in WD. By establishing PPI networks and module analysis of patients with CPS classes A, B and C, six, 13, and 12 key nodes were identified, respectively. The biological process enrichment analysis showed that these nodes were mainly associated with the metabolic process (GO: 0008152), biological regulation (GO: 0065007), and cellular process (GO: 0009987).

The MGDRN topological analysis of AIH patients led to the identification of five key miRNAs: hsa-let-7a*, hsa-miR-1260, and hsa-miR-1275 (downregulated); hsa-miR-21 and hsa-miR-222 (upregulated). The most significantly enriched pathways of the downregulated DEMs transcriptionally regulated by small RNAs were p53 tumor suppressor protein (TP53)-regulated metabolic genes (hsa-let-7a*, hsa-miR-1260) and regulation of methyl-CpG-binding protein 2 (MECP2) expression and activity (hsa-miR-1260, hsa-miR-1275). Upregulated DEMs were enriched in interleukin-1 family signaling, Notch2 intracellular domain-regulated transcription (hsa-miR-21), and PTK6-regulated cell cycle and transcriptional regulation of white adipocyte differentiation (hsa-miR-222).

The p53 tumor suppressor protein (TP53) is known to inhibit cell growth by inducing apoptosis, senescence, and cell-cycle arrest. Our results show that hsa-let-7a is involved in liver disease progression through cell apoptosis induction as the p53 pathway mediator regulated AGO1, AGO2, AGO4, COX14, COX6B1, MT-CO1, MT-CO2, SESN1, SESN2, and YWHAZ.

We selected three of the key miRNAs (hsa-miR-195, hsa-miR-182 hsa-miR-23a) that were significantly differently expressed in alcoholic liver disease. The main pathways of target genes regulated by these miRNAs are regulation of PTEN mRNA translation (hsa-miR-23a), phospholipid metabolism (hsa-miR-195), and post-translational protein modification (hsa-miR-182). Peyrou et al. [25] reported that dysregulated PTEN expression is observed with alcohol consumption and ethanol induces alterations of PTEN expression/activity in the liver; hence, PTEN expression represents a potential common therapeutic target for alcoholic liver disease. Wang et al. [26] indicated that miR-125b, miR-146a, and miR-155 can regulate inflammatory responses to TNF-α in Kupffer cells and that Kupffer cell-specific miR-155 contributes to alcohol-induced activation of TNF-α in macrophages from patients with ALD. Bala et al. [27] pointed out a significant decrease in the expression of hsa-miR-182 and hsa-miR-222 in alcoholic liver disease.

Using topological analysis, we identified four hub miRNAs, namely, hsa-miR-1227, hsa-miR-1275, and hsa-miR-148a (downregulated), and hsa-miR-374 (upregulated), in hepatitis C patients. These miRNAs mainly participate in the regulation of MECP2 expression and activity, interferon alpha/beta signaling (hsa-miR-1227), oncogene-induced senescence, glycosaminoglycan metabolism (hsa-miR-148a), and transcriptional regulation of granulopoiesis (hsa-miR-374). Patients with viral hepatitis are at increased risk of developing cirrhosis and primary liver cancer. Schueller et al. [28] demonstrated that downregulation of miR-29 was observed in patients with HCV-induced fibrosis. Moreover, their results showed that miR-122 contributes to the liver tropism of HCV by increasing the binding of ribosomes to the viral RNA, thereby stimulating HCV translation; thus, inhibition of miR-122 might decrease HCV replication. Janssen et al. [29] showed that Miravirsen, a modified oligonucleotide targeting miR-122, caused prolonged dose-dependent reductions in HCV RNA levels without evidence of viral resistance in patients with chronic HCV genotype 1 infection. Miravirsen is a locked nucleic acid-modified DNA phosphorothioate antisense oligonucleotide that sequesters mature miR-122 in a highly stable heteroduplex, thereby inhibiting its function. Bueno Marinas et al. [30] observed that miR-122-5p was underexpressed in tissue and overexpressed in the circulation, supporting the hypothesis that the high blood levels of miRNAs might be released from apoptotic or necrotic cardiomyocytes, indicating progression of tissue damage. They indicated that myocardial damage is reflected in the circulation and that these miRNAs can be used as noninvasive biomarkers of the disease, permitting differential diagnosis.

The pathway enrichment of hub miRNAs in PBC showed that hsa-miR-23b, hsa-miR-1227, hsa-miR-1275, and hsa-miR-148a (downregulated) are enriched in interleukin-12 signaling, Golgi-associated vesicle biogenesis, interferon alpha/beta signaling, Notch2 intracellular domain regulates transcription, and MAPK6/MAPK4 signaling, while hsa-miR-374b* (upregulated) is involved in transcriptional regulation of white adipocyte differentiation and transcriptional regulation of granulopoiesis. Our results suggest that hsa-miR-23b is involved in pathophysiology of primary biliary cholangitis through interleukin-12 signaling via regulation of CNN2, JAK1, LMNB1, MTAP, SOD2, and TCP1. Padgett et al. [16], investigating liver tissue from six patients with end-stage PBC, found hsa-miR-23b to be lower expressed in PBC versus controls.

The diagnosis of PSC is difficult because of a lack of biomarkers; hence, the discovery of specific biomarkers that can be measured routinely in samples to correctly diagnose PSC is an unmet clinical need. In this study, via topological analysis of primary sclerosing cholangitis patients, we observed a significant downregulation of hsa-miR-23b, hsa-miR-378, hsa-miR-1275, and hsa-miR-148a, demonstrating that reduced expression of these miRNAs may be associated with PSC progression. The result of our pathway enrichment analysis indicated these four hub miRNAs are mainly enriched in the Toll-like receptor 9 (TLR9) cascade, cytokine signaling in immune system, and Wnt signaling pathway. Loosen et al. [31] reported that, in PSC patients, serum levels of miR-1281 and miR-126 were significantly increased compared to healthy controls, and they might, therefore, be useful as biomarkers for the diagnosis of PSC. Moreover, Bernuzzi et al. [32] described miR-200c as significantly downregulated in patients with PSC in a large screening approach including 667 miRNAs.

Wilson disease is an inborn disorder of the copper metabolism that is caused by a defect in the P-type ATPase gene (ATP7B). Impaired function of ATP7B then results in copper accumulation, leading to the hepatic appearance of WD. In WD patients, we observed that hsa-miR-1275 was significantly decreased and hsa-miR-21 and hsa-miR-222 were significantly increased compared to healthy controls. The results of our KEGG enrichment showed that hsa-miR-222 is involved in copper homeostasis by regulating APP, ATP7B, FOXO1, FOXO3, MDM2, PTEN, and TP53. Our results demonstrated that hsa-miR-222 probably has an important role in WD progression through regulating copper homeostasis by binding to the 3′ untranslated region of ATP7B as target genes and then blocking the protein translation.

Our study also provides so far unpublished information about miRNA changes in liver samples stratified according to the organ functional state using the Child–Pugh score. Our analysis of the miRNA–gene differential regulatory network of all patients according to the Child-Pugh score classification identified the following differentially regulated miRNAs: hsa-miR-1275, hsa-miR-29a, hsa-miR-320, hsa-miR-1227, hsa-miR-378, hsa-miR-21, and hsa-miR-182, which could serve as biomarkers for prognosis, diagnosis, and treatment of different stages of liver diseases. All patients with the Child–Pugh score A and B had a significant decrease in expression of hsa-miR-1275 and hsa-miR-320, whereas subjects with advanced stage of liver disease (Child–Pugh score C) showed a higher expression of hsa-miR-21 and hsa-miR-182 compared to the lower Child–Pugh score groups. The downregulated miRNAs (hsa-miR-1275, hsa-miR-1227, hsa-miR-378, hsa-miR-320, hsa-miR-29a) are implicated in regulation of methyl-CpG-binding protein 2 (MECP2, a master epigenetic orchestrator of hepatic stellate cell activation) expression and activity, epigenetic regulation of gene expression, degradation of the extracellular matrix, and peroxisomal protein import. Hardy and Mann [33] reported that liver fibrosis in mice is regulated by an epigenetic relay pathway that includes MECP2, miRNA132, and EZH2. The authors found that MECP2 translation is regulated by miR132, and disruption of MECP2 reduced the fibrogenic features of myofibroblasts and attenuated fibrogenesis in chronic liver disease. Our results show that, in human liver disease, downregulation of hsa-miR-1275 and related target genes might lead to an increase in MECP2 expression andm consequently, progression of liver injury. We also identified another pathway regulated by miRNA related to the functional state of the liver. Our results probably present new insight into the regulation of six genes (PELI1, TIAM1, KRIT1, RBPJ, ZNF367, CPEB3) involved in the Notch signaling pathway by hsa-miR-21, which are related to patients with the Child–Pugh score C. Hence, a key mechanism and a potential target pathway in regulating liver disease is the Notch signaling pathway.

## 4. Materials and Methods

### 4.1. Patient Characteristics

Liver samples were taken from excised organs of Caucasian patients diagnosed according to the standard clinical criteria with hepatitis C (HCV, *n* = 23), primary biliary cholangitis (PBC, *n* = 14), primary sclerosing cholangitis (PSC, *n* = 8), alcoholic liver disease (ALD, *n* = 22), Wilson‘s disease (WD, *n* = 9), and autoimmune hepatitis (AIH, *n* = 16), and subjected to organ transplantation. The stage of liver dysfunction was classified according to the Child–Pugh score. All patients met clinical criteria for transplantation (Child–Pugh class B and C, as well as Child–Pugh class A), presenting elevated levels of α-fetoprotein, unstable blood pressure in the portal vein, or hepatic encephalopathy. Fifteen patients, in addition to liver pathology, suffered from diabetes mellitus type 2, while 13 suffered from arterial hypertension, and three suffered from psoriasis. Mixed origin liver pathologies (e.g., AIH/HCV, ALD/HCV, and AIH/PBC), as well as heart failure, kidney failure, inflammatory, and other autoimmune diseases, constituted exclusion criteria. Characteristics of the subjects are presented in Table 1. The samples of normal liver tissue were obtained from patients without liver diseases undergoing resection of metastasis from colon carcinoma. The normal tissue was located at least 5 cm away from the lesion and did not show any histological alteration, whereas abnormal laboratory blood liver function tests were not measured. All patients were admitted to the Liver Unit of the Department of General and Transplantation Surgery, County Hospital, Szczecin, Poland from 2008 to 2016. Informed consent was obtained from all patients, and the study protocol was approved by the Bioethics Committee of the Pomeranian Medical University (Approval number: BN-001/11/07, Date: February 28, 2007).

### 4.2. Tissue Collection

Tissue fragments were dissected from livers under standard general anesthesia (propofol, sevoflurane, rocuronium, fentanyl, dipyrone) no later than 15 min after blood flow clamping. Samples were immediately immersed in RNAlater (Applied Biosystems, Darmstadt, Germany) and stored at −80 °C for miRNA and total RNA analysis.

### 4.3. RNA Extraction and Real-Time PCR

Total RNA (including small RNA) was extracted from 50 mg tissue samples with Direct-zol RNA Miniprep Plus kit (Zymo Research, Irvine, CA, USA); subsequently, RNA concentration was measured using a NanoDrop ultraviolet (UV) spectrometer (Thermo Fisher Scientific, Waltham, MA, USA). Reverse transcription was performed using TaqMan^®^ MicroRNA Reverse Transcription Kit with Megaplex™ RT Primers, Human Pool Set v3.0 (Thermo Fisher Scientific, USA) and 500 ng of total RNA in a reaction volume of 7.5 µL, separately for each of two pools of miRNA. Finally, quantitative PCR was performed in a ViiA7 Real-Time PCR System using TaqMan Array Human MicroRNA Card Set v3.0 (Thermo Fisher Scientific, USA). This set enables quantitation of 472 previously described human microRNAs and three housekeeping genes as endogenous controls (stably expressed small noncoding RNAs: U6 snRNA, RNU44, and RNU48) to aid in data normalization. The same threshold value was manually set for all targets, and only miRNA assays with Ct values ≤ 32 were further analyzed. The relative quantity (RQ) of each target was calculated using the ΔC_t_ method, in relation to mean expression of three endogenous controls.

### 4.4. Identification of Differentially Expressed miRNA and Prediction of Target Genes

Statistical analysis to explore differentially expressed miRNA (DEMs) between each disease group and control group was performed using ANOVA on the normalized miRNA counts using R version 3.4.4 (http://www.Rproject.org). The Holm–Bonferroni method was used to correct for multiple testing, and miRNAs with adjusted *p*-value ≤ 0.05 were considered DEMs. The target genes of the DEMs were predicted using the miRDB database (http://mirdb.org). We used a heatmap of the expression data to summarize the relationships between liver patient groups at different stage of liver dysfunction (Child–Pugh score A, B, C) and the control group.

### 4.5. Protein–Protein Interaction (PPI) Network Construction and Module Screening

In order to interpret the interactive relationship among DEM gene targets, PPI networks were constructed using the STRING database (http://string-db.org) [34], with the highest confidence threshold (0.900). Each score is derived by benchmarking analysis and generally corresponds to the estimate of how likely a given association describes a functional linkage between two genes.

The result was visualized using Cytoscape version 3.7.1 [35]. The Cytoscape plug-in Molecular Complex Detection (MCODE, http://apps.cytoscape.org/apps/mcode) was employed to analyze modules. The parameters of Cytoscape analysis were set as follows: degree cutoff = 2, node score cutoff = 0.2, k-score = 2, and max depth = 100.

### 4.6. miRNA–Gene Differential Regulatory Network (MGDRN) Construction

To identify liver disease-specific differentially regulated miRNA–mRNA relationships, MGDRNs were built using the SCAN toolbox in Cytoscape. In order to find key miRNAs, some topological properties for each node (degree, betweenness centrality, and closeness centrality) of the constructed networks were calculated using the Network Analyzer plug-in [36]. The node degree corresponds to the number of edges (*n*) linked to a node (*v*), where the degree (*v*) = *n*. Betweenness centrality measures the centrality of each node on the basis of shortest paths between all other nodes, as shown in the following equation:

(1) $$\mathrm{Betweenness}\;\mathrm{centrality}\;\left(v\right)=\sum_{\;s\neq v\neq t}\frac{\sigma_{st}\left(v\right)}{\sigma_{st}},$$

where σ_st_ is the total number of shortest paths from node *s* to node *t*, and σst (*v*) is the number of the paths that pass through node *v*. Closeness centrality measures the mean distance from a node *v* to all other nodes, as shown in the following equation:

(2) $$\mathrm{Closeness}\;\mathrm{centrality}\;\left(v\right)=\frac{1}{\sum_{u=1}^{n}d\left(u,v\right)},$$

where d(u, v) is the shortest distance between node *u* and node *v*, and *n* is the number of nodes in the network.

### 4.7. Functional and Pathway Enrichment Analysis

To understand the potential functional role of key miRNAs in MGDRNs, GO annotation and KEGG pathway enrichment analysis were performed for each miRNA using its respective target genes. Enrichment analysis was conducted using the Database for Annotation, Visualization, and Integrated Discovery (DAVID; http://david.abcc.ncifcrf.gov/). A significance level of α = 0.05 was considered the limit of significance.

## 5. Conclusions

Our findings show a novel panel of deregulated miRNAs in the livers from patients with different liver pathologies, used to investigate their potential as biomarkers for detection in different etiology and staging. In fact, the present work shows that specific patterns of deregulated miRNAs are able to support each specific diagnosis. Development of microRNA panels, either alone or in combination with classical biomarkers, might provide earlier warning of changes in liver function preceding manifestation of some liver diseases, helping to inform treatment decisions, establish prognosis, or improve survival. Our data are the first to present a panel of altered expression of miRNA in liver patients with different etiology and stages of Child–Pugh score. A limitation of our analysis is that only bulk expression data were generated; hence, we were not able to trace expression changes back to different cell types. Furthermore, we provided an analysis of miRNA expression in liver, thus requiring an invasive biopsy. Further studies are needed to evaluate if similar miRNA patterns are also detectable in blood. As only limited clinical follow-up was available for our study participants, the prognostic and clinical impact of the differentially expressed miRNAs in terms of predicting disease progression and survival could not be evaluated in this study; hence, additional studies are needed to evaluate the prognostic value and to clinically validate our results.

## Figures and Tables

**Table 1:** Characteristics of subjects. Patient cohorts are hepatitis C (HCV, *n* = 23), primary biliary cholangitis (PBC, *n* = 14), primary sclerosing cholangitis (PSC, *n* = 8), alcoholic liver disease (ALD, *n* = 22), Wilson’s disease (WD, *n* = 9), and autoimmune hepatitis (AIH, *n* = 16). Other information include the stage of cirrhosis progression according to the Child–Pugh score (CPS), total bilirubin, albumin, prothrombin time, and international normalized ratio.

Parameter/Disease	Control	HCV	PBC	PSC	ALD	AIH	WD
Sex (male/female)	17/16	12/11	7/7	4/4	11/11	8/8	5/4
Age (years)	63 ± 10	52 ± 5	59 ± 4	43 ± 10	51 ± 6	47 ± 16	35 ± 12
Child–Pugh (A/B/C)	-	7/10/4	2/4/4	3/3/0	0/8/12	6/6/8	1/4/4
Total bilirubin (mg/dL)	0.59 ± 0.25	2.38 ± 1.37	6.42 ± 6.72	8.14 ± 8.14	4.4 ± 4.02	3.54 ± 3.53	8.7 ± 10.5
Albumin (g/dL)	3.89 ± 0.38	3.31 ± 0.45	3.13 ± 0.65	3.7 ± 0.44	3.03 ± 0.50	3.29 ± 0.39	3.4 ± 0.67
PT ^1^ (s)	12.7 ± 2.3	14.4 ± 2.0	12.5 ± 1.2	13.2 ± 2.8	16.0 ± 2.2	14.6 ± 2.5	32.1 ± 14.2
INR ^2^	1.14 ± 0.21	1.39 ± 0.27	1.19 ± 0.21	1.4 ± 0.52	1.47 ± 0.23	1.42 ± 0.41	2.7 ± 1.4

^1^ Prothrombin time; ^2^ international normalized ratio.

**Table 2:** The lists of some features of each study group’s protein–protein interaction (PPI) network.

Condition	Number of Nodes	Number of Edges	Average Node Degree	Average Local Clustering coefficient	PPI Enrichment *p*-Value
ALD	281	115	0.819	0.213	7.14 × 10^−5^
AIH	109	27	0.495	0.284	0.194
PBC	253	188	1.49	0.265	1.48 × 10^−12^
HCV	347	277	1.6	0.324	1.64 × 10^−9^
PSC	157	40	0.51	0.123	0.00222
WD	230	87	0.757	0.202	1.72 × 10^−5^
CPS A	237	93	0.785	0.253	0.00561
CPS B	367	233	1.27	0.245	2.46 × 10^−6^
CPS C	376	231	1.23	0.258	2.06 × 10^−5^

**Table 3:** The topological features of the miRNA–gene target differential regulatory network for each condition.

Topological Features	According to Different Etiology							According to Child-Pugh Score		
	Total	AIH	ALD	HCV	PBC	PSC	WD	A	B	C
Number of nodes	1165	137	365	461	327	204	297	442	495	503
Number of edges	1420	125	380	474	323	203	314	477	518	559
Shortest paths (%)	44	9	12	14	7	5	23	7	11	17
Diameter	24	6	17	17	13	6	20	15	22	18
Average neighbors	2.421	1.825	2.016	2.004	1.9202	1.873	2.027	2.036	2.044	2.087
Density	0.002	0.013	0.006	0.004	0.006	0.009	0.007	0.005	0.004	0.004
Centralization	0.024	0.173	0.063	0.050	0.071	0.115	0.078	0.052	0.047	0.046
Heterogeneity	1.088	1.502	1.311	1.239	1.351	1.295	1.415	1.258	1.210	1.205

**Table 4:** Summary of deregulated key DEMs with maximum degree, betweenness centrality, and closeness centrality identified in different conditions of liver disease.

Condition	Upregulated	Downregulated
ALD	hsa-mir-195, hsa-mir-182, hsa-mir-23a	-
AIH	hsa-mir-222, hsa-mir-21	hsa-mir-1260, hsa-mir-1275, hsa-let-7 *
PBC	hsa-mir-374b *	hsa-mir-23b, hsa-mir-1227, hsa-mir-1275, hsa-mir-148a
HCV	hsa-mir-374	hsa-mir-1227, hsa-mir-1275, hsa-mir-148a
PSC	-	hsa-mir-378, hsa-mir-23b, hsa-mir-1275, hsa-mir-148a
WD	hsa-mir-222, hsa-mir-21	hsa-mir-1275
CPS A	-	hsa-mir-1275, hsa-mir-29a, hsa-mir-1227, hsa-mir-320
CPS B	-	hsa-mir-1275, hsa-mir-320
CPS C	hsa-mir-21, hsa-mir-182	hsa-mir-378, hsa-mir-1275

* miRNA denotes passenger miRNA.

## Supplementary Materials

Supplementary materials can be found at https://www.mdpi.com/1422-0067/21/19/7368/s1.
